# Barriers and Facilitators to Implementing a Community-Based Psychosocial Support Intervention Conducted In-Person and Remotely: A Qualitative Study in Quibdó, Colombia

**DOI:** 10.9745/GHSP-D-23-00032

**Published:** 2024-02-28

**Authors:** Diana Carolina Chaparro Buitrago, Michel Rattner, Leah Emily James, Juan Fernando Botero García

**Affiliations:** a Department of Global Health, McMaster University, Hamilton, Canada.; b Department of Psychology, Universidad de los Andes, Bogotá, Colombia.; c Palo Alto University, Department of Psychology, Palo Alto, California, USA.; d Heartland Alliance International, Chicago, IL, USA.; e Heartland Alliance International, Cali, Colombia.

## Abstract

Community-based psychosocial support group (CB-PSS) interventions using task-shifting approaches are well suited to provide culturally appropriate services in low- and middle-income countries. However, contextual barriers and facilitators must be considered to tailor interventions effectively, particularly considering the challenges introduced by the COVID-19 pandemic. We explore the barriers, facilitators, and psychosocial changes associated with implementing a CB-PSS group intervention delivered by local lay providers to conflict-affected adults in Quibdó, Colombia, using both in-person and remote modalities. Data were analyzed from 25 individual interviews with participants and a focus group discussion involving staff members, including 7 community psychosocial agent facilitators and 2 mental health professional supervisors. The analysis used a thematic approach grounded in a descriptive phenomenology to explore the lived experiences of participants and staff members during implementation. Participant attendance in the in-person modality was compromised by factors such as competing work and family responsibilities and disruption caused by the COVID-19 pandemic. Participants in the remote modality faced challenges concerning unstable Internet connectivity, recurrent power outages caused by heavy rain, distractions, interruptions, and threats to confidentiality by family and coworkers. Despite these challenges, data revealed key contextual facilitators, including the community-based knowledge of facilitators and integration of traditional practices, such as the *comadreo* (informal talks and gatherings). Respondents shared that the CB-PSS groups promoted stronger community relationships and created opportunities for participants to exchange peer support, practice leadership skills, develop problem-solving skills based on peers’ experiences, and enhance emotional regulation skills. Differences and similarities across in-person and remote modalities are discussed, as are key considerations for practitioners and policymakers.

## Abstract (precis)

This study explores contextual barriers and facilitators and perceived psychosocial changes associated with implementing a community-based psychosocial support group intervention for conflict-affected adults delivered via in-person and remote modalities and presents recommendations for strengthening the provision of community-based services within routine mental health services in Colombia.

## Teaser

**Key Findings:**

- Barriers to implementing a community-based psychosocial support (CB-PSS) group via the in-person modality included poor attendance and competing activities and via the remote modality included issues with poor connectivity, privacy, and frequent interruptions.
- Including community leaders and lay community providers helped foster acceptability and tailoring to cultural norms and values of the Quibdó population.
- For both in-person and remote modalities, traditional cultural processes fostered the exchange of peer support and the development of psychosocial skills.

**Key Implications:**

- Practitioners should consider integrating cultural practices and norms into CB-PSS groups and leveraging them through facilitation by community lay providers.
- Practitioners should consider the potential to utilize remote modalities to improve access to mental health and psychosocial support services (MHPSS). However, more work is needed to address issues concerning privacy and confidentiality that may jeopardize participant engagement, retention, safety, and benefits gained.
- Policymakers should consider using CB-PSS groups and other task-shifting models to overcome barriers to accessibility of MHPSS where the health system does not have adequate coverage.

## INTRODUCTION

Despite growing evidence supporting the effectiveness of mental health and psychosocial support (MHPSS) interventions, low- and middle-income countries (LMICs) still face significant challenges in ensuring access to these services.^1^ The Sustainable Development Goals and the World Health Organization’s Comprehensive Mental Health Action Program have emphasized the urgency of addressing mental health gaps and disparities.^2^ Nevertheless, evidence suggests that the need for MHPSS services still outweighs the capacity of the social service and health care system in many settings.^3^^,^^4^

In Colombia, an armed conflict that has persisted for over 6 decades has significantly heightened risks to mental health and psychosocial well-being due to serious human rights violations, forced displacement, exposure to combat, threats, torture, sexual violence, forced disappearances, and homicides, as attested by more than 900,000 victims reported in the Victims Unit Registry.^5^^,^^6^ Exposure to conflict violence has been directly linked to a deterioration of social capital and poor mental health, including anxiety, depression, and post-traumatic stress disorder (PTSD) among survivors.^7^^,^^8^ As part of the Colombian peace accord in 2016, a policy framework prioritizing MHPSS services was developed to assist victims of the armed conflict.^9^^–^^11^ These efforts encompass culturally sensitive components, continuous capacity-strengthening, and interdisciplinary coordination, including integration of local lay providers to optimize service provision.^12^^,^^13^ However, despite the Colombian government’s efforts to improve access to MHPSS services, many challenges remain to translate this policy framework into reality.^14^

Barriers to delivery of feasible, acceptable, and effective MHPSS services are widespread, particularly in vulnerable and conflict-affected regions marked by high levels of unemployment, poverty, and limited access to education and health services.^7^^,^^14^^–^^16^ MHPSS service provision is predominantly concentrated in major cities, leaving rural areas significantly underserved.^16^ Those residing in remote locations face substantial challenges due to travel distances and costs.^15^ The scarcity of specialized providers in these areas further exacerbates the situation.^16^ A mixed-method study conducted in Chocó^14^ revealed barriers to service accessibility (e.g., 20% of municipalities lack MHPSS services), acceptability (stemming from stigmatizing beliefs among providers and the community), and insufficient cultural adaptation of services.

The COVID-19 pandemic worsened these barriers while also intensifying MHPSS needs.^17^ Measures taken to control the virus, such as lockdowns, social distancing precautions, and mobility restrictions, unintentionally exacerbated mental health outcomes while also impeding access to already limited services.^17^ In regions like Quibdó, these measures further impeded health care access, forcing reliance on traditional medicine, which was not always the most suitable alternative for treating severe symptoms associated with COVID-19 infection.^18^ This situation was further aggravated in certain areas due to additional mobility restrictions imposed by armed groups. Moreover, due to strict lockdowns, many Afro-Colombians experienced curtailed traditional mourning practices, exacerbating emotional distress.^18^ In Colombia, rates of anxiety increased by 2.5–2.8 times and depression by 1.5–1.9 times.^19^

In response to the unique challenges faced by LMICs, the World Health Organization^20^ has proposed using task-shifting models (TSMs) to address gaps in health service provision. TSMs entail training and handing over activities and services from specialized professionals to paraprofessional personnel or nonprofessionally trained lay providers, typically from the community.^21^ This approach is now widely implemented in LMICs and humanitarian settings.^22^ TSM approaches, such as the Mental Health Gap Action Program^23^ and Problem Management Plus (PM+),^24^^,^^25^ offer low-cost, evidence-based services. These approaches enhance cultural appropriateness by involving nonspecialized health workers and incorporating contextual healing practices tailored to community needs. Additionally, digital interventions, in which services are provided online or by phone, have also emerged as an alternative to overcome the challenges of providing safe and accessible in-person services, especially in the context of the COVID-19 pandemic.^26^ These strategies have shown promising results in reducing COVID-19 exposure, saving costs and travel time, providing scheduling flexibility, and expanding access.^27^ However, many questions persist regarding the feasibility and acceptability of implementing community-based psychosocial support (CB-PSS) groups using TSMs and digital interventions, including in Colombia and during the COVID-19 pandemic.^28^^–^^30^

Many questions persist regarding the feasibility and acceptability of implementing community-based psychosocial support groups using task-shifting models and digital interventions, including in Colombia and during the COVID-19 pandemic.

A mixed-method pilot study^31^ was conducted in 2020 in Quibdó, Colombia, delivered by local community lay providers via in-person, remote, and hybrid modalities. The findings informed the design of the current study, which was conducted as part of a randomized controlled trial (RCT) with both quantitative (results forthcoming) and qualitative elements. The current article describes the results of qualitative analysis designed to explore perceived barriers, facilitators, and psychosocial changes among participants and staff members (lay providers and mental health supervisors) associated with a CB-PSS group intervention delivered via in-person and remote modalities for conflict-affected adults during the COVID-19 pandemic in Quibdó, Colombia. The following questions guided the study:

- What are the key barriers and facilitators associated with implementation of a CB-PSS intervention as perceived by participants and staff members?
- What psychosocial changes were experienced as a result of intervention participation, as perceived by participants and staff members?
- How did barriers, facilitators, and psychosocial changes differ in in-person and remote modalities?

## METHODS

### Project Setting

Quibdó, the capital city of the Chocó department located on the Colombian Pacific coast, is severely affected by armed conflict, narcotraffic, corruption, and poverty.^8^ According to Colombia’s National Planning Department, the incidence of poverty in Quibdó is 64.8% (compared to 39.3% nationally).^32^ Basic needs are often unmet, with only 25.2% of the population having access to clean water, 17.8% to sewer services, and 26.5% living in structural or space-inadequate or overcrowded households.^6^^,^^18^^,^^32^ According to the Internal Displacement Monitoring Centre, in 2021, Chocó was 1 of the 3 primary receptors of internally displaced persons, receiving approximately 213,305 people, of which Quibdó received 60,136.^33^ In addition, of the 1.8 million Venezuelans registered in Colombia’s temporary protection statute, 2,994 reside in Chocó.^34^ Many Venezuelans who were forcedly displaced from their country also suffered direct or indirect effects of the armed conflict in Colombia, including further displacement, violence, and recruitment by armed groups who then forced them to work in coca fields, engage in illegal mining and drug trafficking, and subject them to sexual exploitation.^35^ Moreover, from April to June 2021, during the implementation of this project, the country witnessed its most widespread national strike. Numerous crimes and human rights transgressions attributed to government armed forces were reported, and the economic and material consequences of the strike affected most of the country, including Quibdó.^36^

### Program Description

The current study builds upon the Association of Organizations for Emotional Support (ACOPLE) program funded by the United States Agency for International Development. From 2010 to 2020, Heartland Alliance International (HAI) implemented ACOPLE, with the support of the National Association of Displaced Afro-Colombians, the Institute for Research and Development in the Prevention of Violence and Promotion of Social Coexistence, the Universidad del Valle, and Johns Hopkins University. ACOPLE sought to provide MHPSS to Afro-Colombian communities on Colombia’s Pacific Coast in individual^37^ and group^38^^,^^39^ formats. The program used a TSM in which services were delivered by community psychosocial agents (CPAs) trained as lay providers and supervised by professionals. Lessons learned from ACOPLE’s prior studies include incorporating culturally based components as part of intervention protocols.^38^^–^^41^

In recent years, ACOPLE’s CB-PSS group model evolved based on participant and staff feedback to increase focus on community problem-solving and culturally based expressive activities. The current intervention consists of 8 sessions, including an introductory session and 3 collaborative problem-solving sessions (informed by PM+)^25^ interspersed with 4 expressive sessions based on cultural practices such as dance and artwork, designed to foster emotion identification and regulation (more detail on intervention content is provided in the pilot study article).^31^

### Participants and Recruitment

The current study was conducted as part of a broader RCT (results forthcoming). Adults aged 18 years and older residing in Quibdó and the rural community of Tutunendo (located approximately 15 minutes northeast of Quibdó) who reported exposure to violence associated with the armed conflict were eligible to participate in the RCT. Participants of this qualitative study were purposively selected from the experimental group of the RCT, with clustering based on group modality to ensure a balanced representation of in-person and remote modalities, which constitutes the main focus of the analysis. Additionally, differences in results according to sociodemographic characteristics, including location (rural and urban areas), gender, age, employment status, education level, and nationality, were discussed to capture variations in study participants’ experiences.

After recruitment, participants could choose to join either an in-person modality (with sessions held in community centers adhering to biosecurity protocols) or a remote modality (with online sessions facilitated via the Zoom platform). In total, 165 participants chose to participate in person (making up 10 groups), and 103 participants participated remotely (making up 8 groups). Each group had 7 to 10 participants. Participants were then randomized to experimental (n=134) and waitlist control groups (n=134).

From these clusters, 25 participants were randomly selected for individual semistructured interviews. Table 1 illustrates the sociodemographic characteristics of the individuals interviewed. All staff members (n=10) were invited to participate in a focus group discussion (FGD). Nine participated, including 7 CPAs and 2 professional supervisors (1 psychologist and 1 social worker). Table 2 provides information concerning the sociodemographic characteristics of staff members in the FGD. Five participants and 1 CPA could not participate due to conflicting activities.

**TABLE 1.** Sociodemographic Distribution of Community-Based Psychosocial Support Group Participants Interviewed, Quibdó, Colombia

**Variables**	**In-Person,** **No. (%)(n=15)**	**Remote,** **No. (%)** **(n=10)**
Gender		
Men	5 (33.3)	2 (20)
Women	10 (66.7)	8 (80)
Age, median (SD; range), years	56.1 (16.2; 22–75)	34.8 (12.8; 18–63)
Ethnicity		
Afro-Colombian	13 (86.7)	6 (60)
Indigenous	0 (0)	1 (10)
Other	2 (13.3)	3 (30)
Nationality		
Colombian	12 (48)	7 (28)
Venezuelan	2 (8)	3 (12)
Dual citizenship (Colombian and Venezuelan)	1 (4)	0 (0)
Location		
Urban	11 (73.3)	8 (80)
Rural	4 (26.7)	2 (20)
Education level		
Primary school or less	9 (60.0)	1 (10)
Middle to high school	4 (26.7)	4 (40)
Undergraduate degree or higher	2 (13.3)	5 (50)
Marital status		
Single	3 (20)	4 (40)
Married/in a relationship	11 (73.3)	6 (60)
Widowed	1 (6.7)	0
Employment status		
Formal	0 (0)	1 (10)
Informal	9 (60)	3 (30)
Work at home	1 (6.7)	5 (50)
Student	0 (0)	1 (10)
Unemployed	5 (33.3)	0 (0)
Internally displaced		
Yes	10 (66.7)	8 (80)
No	5 (33.3)	2 (20)
Traumatic events, median (SD; range) (7 max)	2.07 (1.6; 0– 7)	1.60 (1.4; 0–7)

Abbreviation: SD, standard deviation.

**TABLE 2.** Sociodemographic Characteristics of Focus Group Discussion With Staff Members, Quibdó, Colombia

**Variables**	**No. (%)**
Gender	
Woman	9 (100)
Age, years	
30–39	5 (56)
40–49	4 (44)
Ethnicity	
Afro-descendant	9 (100)
Education	
High school	2 (22)
Technician	4 (44)
Undergraduate	1 (11)
Graduate	2 (22)
Marital status	
Married/cohabitation	6 (67)
Single	3 (33)
Job position	
Community psychosocial agent	7 (78)
Psychologist	1 (11)
Social worker	1 (11)
Experience in Heartland Alliance International, years	
1–3	1 (11)
4–6	3 (33)
7–9	5 (56)
Victim of armed conflict	
Yes	7 (78)
No	2 (22)

In-person participants received travel funds sufficient for non-public transport expenses to reduce their exposure to the COVID-19 virus. Remote participants received phone or Internet credit and could borrow smartphones if needed. Service providers completed safety planning checklists with remote participants to promote confidentiality (e.g., to prevent being overheard by household members or coworkers). Both groups were provided with snacks and necessary supplies for each group session. For remote participants, these were delivered to their homes. Groups were facilitated by 8 CPAs working in pairs with 2 professional supervisors (a psychologist and a social worker). All facilitators were members of the target community and had prior experience implementing ACOPLE programming.

### Data Collection and Analysis

One of the coauthors (MR) and another trained professional psychologist (both of whom were not involved in group facilitation) conducted qualitative interviews and the FGD between July and August 2021. During the pilot study,^31^ all interviewing guides and the methodological analytic design were pretested and adjusted for the current study. Two instruments for qualitative data collection were used: (1) an individual semistructured interview topic guide of 16 open-ended questions and (2) an FGD guide consisting of 7 activities designed to facilitate discussion about staff experiences during implementation. During the activities, participants were divided into 2 groups where they discussed and created a visual representation, using a board depicting barriers and facilitators for implementation. Subsequently, each group presented and integrated points. These tools were organized in line with an implementation science framework to explore recruitment, feasibility, commitment, adaptability, utility, scalability, satisfaction, and adoption.^30^^,^^31^^,^^42^ All activities were conducted in Spanish, audio-recorded, transcribed verbatim, and complemented with notes taken by the interviewers.

Fifteen face-to-face interviews were held with participants in the in-person modality and 10 online interviews with participants in the remote modality, each lasting approximately 1 hour. The sample size for each modality in this study was determined based on the recommended number for phenomenological design, from 7 to 10 participants per condition, to capture the in-depth experiences of participants.^43^ Six additional in-person interviews were conducted with participants from the rural community of Tutunendo to explore unique elements of the intervention within this context. The FGD with staff members was conducted in person and lasted approximately 3 hours. To ensure participants’ confidentiality, all the activities were conducted exclusively in the presence of interviewers and participants. All participants were read an informed consent script stating they could withdraw at any point without explanations or penalties or decline to answer any question. All participants provided oral informed consent. No names were collected, and all transcripts were identified with an alphanumeric code number to guarantee confidentiality. The study’s findings were shared with the community, stakeholders, and policymakers.

All activities were analyzed using NVivo v.12 software. Two coders conducted the analysis following a thematic analysis approach based on descriptive phenomenology.^44^ In the initial round of codification, we followed the implementation science framework to organize participants’ experiences into specific categories (Table 3). Subsequently, these descriptive categories were inductively recodified to uncover underlying meanings and patterns within participants’ experiences, resulting in the development of analytical themes and subthemes.^44^ Finally, these themes were revised, refined, and updated according to emerging meanings and codes. The data analysis was conducted in Spanish, and the presented quotes were translated into English and reported according to group modality (i.e., in-person or remote).

**TABLE 3.** Implementation Science Initial Descriptive Categories of Participants’ and Staff Members’ Experiences in CB-PSS Groups

**Category**	**Description**
Commitment	Factors that contributed to participants’ motivation and engagement in the CB-PSS
Feasibility	Factors that facilitate or limit participants attendance to the CB-PSS
Adaptability	Cultural adaptability of the CB-PSS groups
Utility	Effectiveness of the CB-PSS groups to address participants’ psychosocial needs
Scalability	Potential conditions for scaling up the CB-PSS to other demographic populations
Satisfaction	Participants’ satisfaction with the implementation of the CB-PSS
Adoption (only staff members)	Willingness of staff members to adopt the model of the CB-PSS

Abbreviation: CB-PSS, community-based psychosocial support.

### Ethical Approval

The Institutional Review Boards of HAI and Universidad de Los Andes approved the study protocol.

## RESULTS

From the implementation science initial descriptive categories, 4 main themes emerged: (1) contextual barriers, (2) contextual facilitators, (3) support strategies, and (4) psychosocial skills changes and satisfaction (Table 4). We present the results according to the emergent analytical themes and their respective subthemes.

**TABLE 4.** Analytical Themes and Subthemes That Emerged From Analysis of Participants’ and Staff Members’ Experiences in CB-PSS Groups

**Themes and Description**	**Subthemes**	**Description**
**Contextual barriers**: Barriers within the local context that hindered the implementation of the CB-PSS groups.	Participants’ competing responsibilities, COVID-19 infection, and national strike	Participants’ conflicting responsibilities (job, household chores, and academic commitments), as well as the COVID-19 infection, hindered the in-person attendance. None of the participants reported being affected by the national strike.
	Access to technological devices and connectivity	Participants’ limited access to technological devices and connectivity challenges.
	Confidentiality concerns	Factors that compromised confidentiality.
	Acculturation challenges among Venezuelans	Adaptation challenges for Venezuelans.
**Contextual facilitators**: Factors within the context that facilitated the implementation of the CB-PSS groups.	Community organizations and leaders	Local community organizations, local leaders, official institutions demonstrate openness to networking, space facilitation, and promoting the CB-PSS.
	Integration of cultural practices	Incorporation of cultural practices to facilitate the implementation of the intervention.
**Support strategies**: Strategies implemented by the project managers to mitigate contextual challenges and capitalize on contextual facilitators.	Participant engagement and retention strategies	Strategies implemented by the staff members to foster participants’ engagement and retention.
	Strategies to support facilitators	Strategies implemented by project managers to mitigate staff members’ burnout and facilitate peers’ exchange of experiences, resources, and information.
	Use of local knowledge and multicultural perspectives	Community psychosocial agents’ local expertise and cultural openness to integrate diverse participants’ backgrounds.
**Psychosocial skill changes and satisfaction**: Perceived psychosocial skills changes facilitated by the CB-PSS groups.	Community-building skills	Community support skills are cultivated within the groups and exhibited by participants in group sessions and daily lived experiences.
	Problem-solving	Participants enhance skills when discussing problems, suggesting solutions, and implementing them daily.
	Emotional regulation	Participants’ ability to understand, manage, and modify emotions to adapt to different situations and social contexts.
	Satisfaction	Participants’ and staff satisfaction with the implementation of the CB-PSS group activities.

Abbreviation: CB-PSS, community-based psychosocial support.

Despite exploring the rural and urban participants’ experiences, this study did not identify significant differences between the 2 contexts. Nevertheless, some nuanced differences in sociodemographic characteristics, including gender, age, employment status, education level, and nationality, were presented.

### Contextual Barriers

Respondents identified contextual factors that limited accessibility, engagement, and retention in both modalities.

#### Participants’ Competing Responsibilities, COVID-19 Infection, and National Strike

In the in-person modality, predominantly male participants highlighted the challenge of traveling a considerable distance from their homes or workplaces to the group venues that interfered with their participation in scheduled activities. Many participants, particularly women, from this modality recalled missing sessions due to COVID-19 infection, household chores, and taking care of their children and relatives. Both female and male participants cited missing sessions due to medical appointments, travel to other areas, academic commitments, and last-minute work commitments, which is particularly important given that a significant proportion of participants reported informal employment or unemployment.

*We must work hard here, so there were times when I had to work, and couldn’t attend [the session]. Also, I got COVID and got very sick. While I was at home with COVID, I thought, “Oh, but I need the meetings.”* —In-person participant

According to participants, the national strike was not a determining factor limiting them to attend the activities.

#### Access to Technological Devices and Connectivity

In the remote modality, participants shared that they faced limited access to technological devices in their daily lives. The project provided access to a smartphone library, but unexpected technological challenges persisted due to unstable Internet connectivity and power outages caused by heavy rain. Consequently, reconnecting often took a considerable amount of time, leading to frustration and a loss of interest.

In the remote modality, participants shared that they faced limited access to technological devices in their daily lives.

*You were about to answer the question, and felt that urge to participate, but the [Internet] connectivity made it hard to understand. So, that discouraged me a lot because there are important things that make you feel inspired, and I don’t know, you have your time to talk about the topic.* —Remote participant

Furthermore, an elderly female participant highlighted challenges related to technological literacy, attributing them to a lack of formal education and proficiency in using the Internet. To overcome this obstacle, the participant required assistance from a family member and the CPAs, who guided her through the connection process, ultimately facilitating her participation in the sessions.

*Well, since I do not know how to read, my daughter-in-law was the one who helped me connect and dial the phone number. I do not know; she helped me a lot, and so did the CPAs.* —Remote participant

In addition, some participants reported that they connected while at work or commuting back home on public transportation, resulting in joining late, distractions, and disconnections. Participants also described distractions due to family members’ intrusions, social media, and other factors.

*Sometimes, during the meetings, your child might start crying, or well, you become distracted; perhaps your cell phone rings or something, and you find yourself checking your WhatsApp messages, and everyone is like, “blablabla,” and before you realize it, you have missed the whole meeting.* —Remote participant

#### Confidentiality Concerns

The CPAs raised concerns regarding confidentiality and security in the remote modality due to potential for the group content to be overheard by others in the vicinity. Despite CPAs instructing participants to join calls from a private space, not all participants followed these instructions. The CPAs explained that, for comfort and bandwidth reasons, they had given participants the choice to keep their cameras on or off, making monitoring their engagement and surroundings difficult, including whether they were alone during the sessions or if others could overhear them. This sentiment was echoed by a participant who mentioned joining sessions from their workplace.

*Even my boss, where I work, asked me if she could join the sessions. I asked why she was interested, and she explained that she liked the talks. There were times when I was [at work], and she was listening [to the sessions].* —Remote participant

As a result, some participants felt uncomfortable sharing information because they did not know who might be listening. Ultimately, this discouraged participation, as a CPA noticed during the FGD.

*Also, you are worried about safety in some areas, as you never knew who was listening… issues concerning security, so people cannot talk.* —Staff, FGD

In addition, a common dilemma in both modalities was the presence of family and acquaintances within the same group. Although some participants welcomed the idea of including family members in the CB-PSS groups to strengthen community bonds and foster a sense of belonging, the CPAs referred other participants’ concerns about feeling uncomfortable, intimidated, or worried about the potential to jeopardize privacy, heightening stigma and causing disputes and violence.

A common dilemma in both modalities was the presence of family and acquaintances within the same group.

*[…] Not the same family members, but from the same community. So that stops some people from talking because of confidentiality issues; they may feel that something they say might get repeated outside the group.* —Staff, FGD

#### Acculturation Challenges Among Venezuelans

In addition, Venezuelan participants mentioned challenges related to understanding Colombian cultural practices, codes, and dynamics.

*You are aware that I come from a different culture. It is a culture very distinct from this one, and, of course, there are things I cannot even grasp, like their sayings, and what they talk about – do you follow me? Everything here is entirely different. It takes some time to adjust to their ways.* —Remote participant

### Contextual Facilitators

Despite these challenges, important facilitators helped to increase accessibility, engagement, and retention.

#### Community Organizations and Leaders

Staff and participants shared that local organizations and community leaders were instrumental in supporting the implementation of the intervention. Official entities, such as clinics, schools, community centers, and leadership community groups tailored to specific collectives (e.g., Venezuelan association, youth, mental health support, victims’ groups, and WhatsApp community groups), were open to and enthusiastic about connecting community members to services. These organizations facilitated contact, maintained participant engagement, and provided referrals. One Venezuelan participant mentioned the support received from their organization in accessing CB-PSS groups.

*Through the Venezuelan Association in Quibdó, they chose 40 people to help them, that is, to offer [mental health] support.* —Remote participant

#### Integration of Cultural Practices

A salient contextual opportunity facilitating participant engagement and retention was the use of traditional practices (e.g., diverse forms of traditional healing, medicinal plants, sharing of culturally valued food, adherence to community customs, and respect for cultural codes). A specific traditional community practice known as *comadreo* was integrated into the groups and identified as especially important. This practice refers to informal community gatherings where people, often women, come together to engage in conversations, share their daily life experiences, and nurture bonds between community members. The staff shared that incorporating this fundamental component of community life into the groups played a crucial role in helping participants understand and “buy in” to the value of psychosocial support and increasing their willingness to express and share their experiences with their group members.

*[…] People had the opportunity to speak as they do in the comadreo. Comadreo is a traditional practice in which people sometimes find solutions to their problems. The term comes from when neighbors gather at a place, and they are washing and talking, for example, “Oh, comadre [neighbor], this and that happened.” And that is like a way of venting by talking about things, for me, it seems that traditional practices are considered since people had the opportunity to talk to each other about difficult situations.* —Staff, FGD

### Support Strategies

#### Participant Engagement and Retention Strategies

In both modalities, the CPAs’ follow-up phone calls or text messages were crucial in ensuring participants’ motivation and keeping them informed. The CPAs used these communication strategies to remind participants about upcoming sessions, provide support when needed, and follow up with those who had not attended sessions. Several participants expressed their appreciation for this individualized support from the CPAs.

In both modalities, the CPAs’ follow-up phone calls or text messages were crucial in ensuring participants’ motivation and keeping them informed.

In the in-person modality, participants highlighted the importance of biosecurity measures in supporting retention. The implemented protocols, including social distancing, face masks, and handwashing, were consistently followed, making participants feel secure. Notably, 2 elderly participants, 1 female and 1 male, highlighted the substantial impact of being fully vaccinated and the adherence to biosecurity protocols on their perceived safety against potential contagion. This emerged as a crucial factor in maintaining their motivation and ensuring attendance, as 1 participant expressed.

*[…] Well, I am vaccinated. I do not know if they [group members] are vaccinated, but I am vaccinated. So, I took my distance as the [CPAs] told us.* —In-person participant

Additionally, participants expressed appreciation for receiving transportation stipends and refreshments during sessions because many lived far from where the activities took place.

For the remote modality, participants appreciated that CPAs consulted with the group to determine the best times to hold the meetings and were generally flexible with scheduling. Additionally, participants highlighted that providing mobile data to ensure Internet connectivity and the option to borrow phones from the organization allowed them to attend the sessions.

*[…] I didn’t have a phone with enough capacity for Zoom, so they gave me a phone, and in the end, I was able to attend, I think I missed the first 2 sessions … because of the phone. But they gave me one, and I was able to complete the process.* —Remote participant

In contrast, some strategies implemented by the team were either not mentioned or were identified as unhelpful. For example, initially, there was a policy to reschedule sessions if fewer than half of the participants attended. This approach resulted in frustration for those participants who did attend, and as a result, some decided not to return.

*[CPA quoting a participant] “It turns out that I go 2, 3, or 4 sessions, and they send me back home without doing anything.” So, at the fifth session, it turns out that the participant was no longer interested, saying, “I do not want to go, or I do not feel like it anymore, or I do not have time.”* —Staff, FGD

Consequently, the policy was revised so that group sessions were no longer rescheduled regardless of attendance.

Moreover, the team also anticipated confidentiality and privacy concerns in remote groups and conducted a safety planning checklist before the groups started. However, neither participants nor CPAs mentioned its utility. Nevertheless, participants did recall an ongoing emphasis on the importance of confidentiality.

*At the beginning of the sessions, there were some rules: to have the mobile muted, not share information with outsiders, avoid external hearers, remain in a secure place as your own room, and well… do not tell anyone else what was discussed; everything remains within the group.* —Remote participant

#### Strategies to Support Facilitators

The CPAs expressed experiencing burnout due to ongoing challenges such as keeping participants engaged, making decisions about canceling sessions, and tracking participants’ attendance. They shared the importance of regular group supervision sessions and access to HAI’s staff care resources, which included individual counseling sessions with external psychologists facilitated by the organization and online peer support sessions. These resources proved to support their professional capacities and alleviate the emotional distress related to the activities. Additionally, the weekly supervision and team planning sessions were vital spaces for knowledge exchange, sharing experiences, and addressing questions and doubts.

*I believe that the most important training occurred during our weekly planning sessions because that is where we prepared for the day’s session, like role-play […] We do not carry the burden alone; we talk about it, and fortunately, since we have a team and a group, we know how to support each other even when facing difficulties.* —Staff, FGD

#### Use of Local Knowledge and Multicultural Perspectives

Respondents also noted that the CPAs’ experience as local leaders enabled them to harness community opportunities and to effectively adapt CB-PSS activities to meet the participants’ needs and priorities.

*[…] we are aware that, not everyone understands [some concepts], so we have to communicate in a way that people understand easily, but since we know how to do it, we know with whom we are going to work, how to speak to them; then we employ the same dialogue because we are not going to change it, but we try to accommodate it so that the person understands.* —Staff, FGD

Likewise, another CPA shared that her previous experience in providing MHPSS services equipped her with the knowledge and tools needed to overcome obstacles in group facilitation. In practice, the CPAs combined the theoretical and evidence-based psychosocial support skills gained through their training sessions with HAI’s MHPSS technical team during the ACOPLE program’s implementation, along with their own work experience within the communities.

*Our previous experience helped us a lot because a new person may not have the same skills and strategies we do because we learned things at ACOPLE that were useful in our current work.* —Staff, FGD

Furthermore, CPAs facilitated the empowerment of Venezuelan participants, allowing them to assert their voices and promoting the multicultural integration of traditional practices and customs within the group. One Venezuelan participant recalled the opportunity to share their cultural practices, emphasizing the importance of these contributions in fostering community bonds and a sense of belonging within the groups.

*Yes, indeed. Even 3 Venezuelan women participated, and we discussed traditions, foods, and typical dishes. Everyone contributed to the conversation on these topics. […] This helped us express ourselves, and it is wonderful because it fosters sharing, closer relationships, and a stronger sense of camaraderie within the group.* —Remote participant

### Psychosocial Skill Changes and Satisfaction

#### Community-Building Skills

Respondents shared that across both modalities, group activities helped participants cultivate relationships, foster community resources, and translate learning into their interactions with their families and community. Notably, for the in-person modality, participants expressed their appreciation for creating a collaborative environment that fostered a sense of belonging, overcoming mental health stigma, finding support, and a more profound meaning derived from helping others.

*In a community, for example, there are many people, among them, some may be in greater need than others. If I can help someone in solving a problem, something that someone needs, then I am doing something good for the community […]* —In-person participant

Likewise, participants in the remote modality also valued building friendship ties and exchange of peer support, as indicated by 1 participant.

*No one ever imagines discussing infidelity, but I felt secure, and I talked about my situation, and the other girl did too. We all understood each other; what happened to me … I supported my peer, and she supported another one, and so on.* —Remote participant

These views were echoed by a CPA, who highlighted that in both modalities, the CB-PSS groups facilitated the development of stronger relationships and mutual respect among community members.

In both modalities, the CB-PSS groups facilitated the development of stronger relationships and mutual respect among community members.

*The relationship that we have managed to forge in the community since we have found groups where there are people who, despite living in the same community, do not have that friendly interaction with their neighbors. But sometimes there are conflicts and things like that; they begin to have new friends or strengthen their relationships, or at least, they begin to respect others or see them as people who have problems too and that have suffered, as has happened to me.* —Staff, FGD

A community leader participant shared that the groups provided an opportunity to improve their leadership skills. As such, the groups helped him acquire new knowledge and apply it to benefit his community, thus increasing community ownership of the CB-PSS groups and contributing to their scale-up.

*I was motivated because truth be told, I am a community leader, and with each meeting, each session, I gain more knowledge to continue contributing to the community […] So, it is no longer only you are working [HAI], but other than you, each of us … that is why I emphasized community work. That the community can take the initiative to start working in its own space.* —In-person participant

#### Problem-Solving

In both modalities, participants also reported that CB-PSS groups allowed them a platform to openly discuss their problems and collaborate on finding solutions. Some participants emphasized that discussing sensitive issues was initially challenging, but the groups eventually came to be viewed as nonjudgmental environments where they could share their emotional burdens. Participants in the in-person modality appreciated the opportunity to learn from others who had faced similar problems. They also noted that the emotional regulation, communication, and conflict-resolution skills they acquired in the groups helped them address problems and improve their relationships within their families and communities.

Participants in the remote modality, particularly women, also valued the opportunity to vent and exchange problem-solving strategies.

*[…] it does help a lot, you know, to vent and have someone listen to you, not just get back from work and get home to sleep, but it was a space where you could talk about your problems. It may not solve your financial problems, but at least [the CPAs and group members] provided ideas and helped you calm down while exploring alternative solutions.* — Remote participant

#### Emotional Regulation

Across modalities, participants stated that the groups helped them recognize their emotions and learn strategies to regulate them (e.g., relaxation techniques, venting, and expression). In practice, their ability to manage their emotions resulted in improved moods and feeling more relaxed, calm, and positive. These emotional changes were associated with flexible and focused thinking (being mindful, realistic, and task-oriented). Participants in both modalities shared that they learned how to calm themselves down, reducing the likelihood of making impulsive, emotion-driven decisions, which, in turn, facilitated adaptive problem-solving.

*I start reflecting, “If I am about to yell, do not yell, relax, count to 10, and meditate” and so on […] Well, let us take things calmly and think before saying anything; we must think before we act and not act before we think. Because thinking before we act leads to better outcomes.* —Remote participant

#### Satisfaction

Overall, participants expressed high satisfaction with the CB-PSS groups in both modalities. Specifically, the work and support provided by the CPAs were identified as a critical element that contributed to participants’ satisfaction with implementing the activities. In addition, sharing with peers and CPAs helped to foster community bonds that enhance satisfaction. Similarly, CPAs emphasized that witnessing positive changes in participants was their primary source of gratification. They noted that several participants expressed gratitude, highlighting the reciprocal, friendly, kind, and empathetic bond fostered with participants. The CPAs were also satisfied with the activities, training, and supervision processes.

## DISCUSSION

The current study explores contextual barriers and facilitators and perceived psychosocial changes associated with implementation of a CB-PSS group intervention delivered via in-person and remote modalities for conflict-affected adults during the COVID-19 pandemic in Quibdó, Colombia.

### Barriers

It is widely held that the success of a group MHPSS intervention is largely dependent on creating cohesiveness and ensuring safety and privacy among members, which can be highly impacted by attrition, inconsistency in attendance, and confidentiality threats.^45^^,^^46^ Competing activities, including cancellation due to last-minute work shifts, errands, or family demands in the in-person modality, and divided attention due to attending groups while doing other activities and limited digital literacy, especially among older generations in the remote modality, compromised perceived consistency, cohesiveness, and sense of security for group members. These findings are consistent with other work in LMICs, showing that high participant attrition rates, varying job opportunities, and personal and familial demands, such as women often positioned as caregivers within the household, are primary barriers to implementing mental health services and ensuring retention. ^41^^,^^47^^–^^49^

Several strategies to overcome attendance barriers were implemented in the present study, including scheduling adjustments, sending phone and text message reminders, providing instructions for connecting to Zoom, and offering transport and data stipends, in line with strategies used in other research.^46^ However, attempts to mitigate these barriers failed in many instances due to contextual factors (i.e., informal jobs or the difficulty of finding suitable childcare arrangements). Attendance barriers became a primary source of staff burnout. These findings echo other research conducted in the region,^38^^,^^39^ highlighting the sociocontextual challenges in implementing sustainable approaches that may compete with the target population’s unstable work-related opportunities and responsibilities.

Attempts to mitigate attendance barriers failed in many instances due to contextual factors.

Moreover, concerns regarding privacy and confidentiality posed additional barriers, especially in light of mental health stigma.^49^ Previous studies in the region demonstrate that people may experience shame, guilt, and even trauma if their enrollment in mental health services becomes public.^38^^,^^50^ Strategies, such as establishing group rules to ensure confidentiality and privacy and regularly recalling those rules in every session, were implemented in the CB-PSS groups.^46^ Moreover, a safety planning protocol and checklist were used with remote group members to identify private spaces for joining sessions. Nevertheless, respondents reported instances of breaches where family, neighbors, and coworkers intruded on or overheard sessions, compromising the development of a perceived safe space and participants’ willingness to disclose personal information.^48^^,^^51^ More work is needed to develop effective protocols for remote modality to ensure digital literacy, especially for older generations^52^ or those with limited educational levels, and to promote private participation in remote sessions, particularly in settings where crowded living conditions may make this problematic. This is particularly critical for MHPSS interventions in which participants may disclose sensitive content.^53^

Relatedly, some participants perceived attending groups with family or community members as a threat to confidentiality, while others found it to be a source of support for cultivating stronger relationships and a sense of belonging. Questions around facilitation of groups that include acquaintances present a dilemma for community-based MHPSS interventions. Including known community members in the same group may compromise privacy and safety and impede participant sharing of sensitive information. However, if groups do not include community members who live in the same vicinity, participants may not access potential benefits, such as sustainable improvement in community efficacy and cohesion.^54^ Still, to ensure participant safety and comfort, providers may consider offering access to individual services for those who prefer them.

Finally, barriers such as communication interruptions (unstable Internet connectivity) and frequent power outages (blackouts) associated with heavy rain disrupted the continuity of remote groups. Although participants were given data stipends and allowed to borrow smartphones, infrastructural problems in Quibdó significantly impeded implementation. As a result of the COVID-19 emergency, researchers have advocated for mandating governments to prioritize Internet access as a basic need and human right to ensure public access to telehealth services.^55^ While the Colombian government made efforts to improve connectivity in Chocó during the pandemic and access to devices increased during this period, stable Internet connectivity remains a significant challenge in this region.^56^ These barriers continue to challenge equitable implementation of remote services in Colombia and other LMICs and must be considered when planning and implementing interventions in this and similar contexts.^57^^–^^59^

### Facilitators and Support Strategies

Analyses revealed that community leaders and organizations played a key role in fostering acceptability and supporting recruitment and retention of participants, which is consistent with previous research studies.^53^^,^^60^ Moreover, involving CPAs with training and experience providing MHPSS services as facilitators allowed for adaptation to community needs and cultural practices while also maintaining fidelity to the model.^6^^,^^53^^,^^61^ As a result, the intervention effectively blended cultural codes and practices with evidence-based components (i.e., PM+). A notable example of this integration is the incorporation of the comadreo practice, which fostered community support and bonding among group members, creating a space for the inclusion of multicultural elements, such as input from Venezuelan participants. The CB-PSS groups harnessed this familiar concept to enhance participants’ understanding of the value of psychosocial intervention and willingness to engage in exchange of peer support, fostering alliances, trust, and respect among group members. This practice resonates with prior research that emphasizes the significance of Afro-Colombians’ traditional collective practices for recovering social identity, rebuilding networks and relationships, and ultimately alleviating mental health conditions such as anxiety, depression, and PTSD resulting from violence.^6^^,^^39^^–^^41^ Furthermore, it aligns with a broader body of literature that underscores the ability of lay community workers to integrate cultural values, practices, and preferences into MHPSS interventions.^22^^,^^54^ For example, in a series of studies conducted with Latina survivors of intimate partner violence,^62^^–^^64^ trained community workers incorporated the use of common values, cultural codes, shared experiences, and sense of connectedness among members as key elements for collectively restoring ownership of life goals and healing from trauma.^54^^,^^62^^–^^64^

The intervention effectively blended cultural codes and practices with evidence-based components.

As in many LMICs, the COVID-19 pandemic significantly impacted the accessibility of services in Colombia, and consequently, promotion of creative means to ensure equitable access, especially among vulnerable populations, is considered a critical component of ethical and feasible implementation.^27^ Results suggested that providing the option to participate remotely with an Internet stipend and an in-person option with a transportation stipend and biosecurity protocols were highly valued and interpreted as indicative of a caring and accommodating attitude from the implementation team and the organization to overcome challenges such as the national strike. Moreover, frequent follow-up from the CPAs, including phone calls and text message reminders, scheduling adjustments, follow-up with participants who missed sessions, and providing referrals to specialized mental health services for those who required them, played a crucial role in maintaining engagement, developing trust, addressing challenges to retention, and ensuring satisfaction with the intervention, especially given the competing daily life pressures.

However, staff did report experiencing symptoms of burnout, especially associated with participant attendance challenges. These findings align with other research showing that providers frequently grappled with high work demands, emotional exhaustion, loss of perceived self-efficacy, and a reduced sense of personal accomplishments during the pandemic.^65^ The CPAs shared that weekly group supervision sessions were valuable for knowledge exchange and preparation for upcoming sessions. These sessions also served as a platform for team building, offering a safe space for venting, exchanging mutual support, and collaboratively addressing the distress caused by work activities, which resonates with previous research stressing the significance of promoting learning through collective efforts.^66^ Staff appreciated this space to collaboratively develop solutions to problems and enhance their team’s cohesiveness. This approach aligns with an apprenticeship model of supportive supervision,^67^ where supervisors provide weekly in-person and remote supervision and provide constructive debriefing of sessions to support both program quality and staff well-being. Some staff members also highlighted the importance of additional staff well-being support provided by the organization, such as individual sessions facilitated by external providers. The importance of these supports is echoed by other studies,^65^^,^^68^ demonstrating the benefits of promoting emotional awareness, self-care practices, team and organizational support, and individual mental health services and supportive supervision to prevent burnout and promote well-being among community health care providers in humanitarian settings.

### Psychosocial Skills Changes

Participants and staff reported that the CB-PSS groups promoted the cultivation of community-building skills among participants, as well as benefits in problem-solving and emotional regulation skills. Participants’ collective knowledge facilitated the creation of shared approaches tailored to their experiences. These findings align with research using PM+ interventions among conflict-affected individuals and vulnerable adults, delivered by lay providers and professionals in individual^69^ and community^24^ formats. Although research has demonstrated the effectiveness of PM+ in mitigating PTSD, anxiety, and depression and improving well-being and social support in several LMICs,^24^^,^^69^ challenges arise in achieving similar results in settings where building community skills is challenging. For instance, a PM+ intervention in a community group format with Syrian refugees in Jordan camps^70^ failed to demonstrate sustainable improvements in mental health symptoms. The authors hypothesized that the difficult conditions in the camp environment hindered the development of sustainable community support, problem-solving resources, and emotional regulation skills to cope with adversity. The contrasting findings demonstrated the foundational value of community support in grounding the development of other skills (e.g., emotional regulation and problem-solving) within the group formats.

Participants and staff reported that CB-PSS groups promoted the cultivation of community-building skills among participants, as well as benefits in problem-solving and emotional regulation skills.

Participants of this study reported that group discussions focused on emotional coping helped them validate and normalize emotional reactions. This, in turn, strengthened their emotional regulation skills, reducing the likelihood of acting out, improving decision-making processes, and coping when faced with challenging situations. Furthermore, interventions in trauma-affected settings, grounded in solid common elements (i.e., hope and expectancy for change, confidentiality, giving praise, psychoeducation, rapport building, empathy, coping mechanisms, and collaborative goal setting),^71^ have demonstrated the value of promoting common underlying emotional regulation skills to overcome multidimensional mental health consequences.^72^^,^^73^

### Comparative Findings With Partnered Studies

The findings presented here can be considered alongside the results of a pilot study^31^ conducted before the current study. While many results are consistent, in the pilot study, respondents identified a more “collaborative style” in in-person groups than in remote groups. In the current study, a collaborative, peer-support dynamic was identified in both remote and in-person modalities. It is possible that CPAs improved their facilitation skills and were thus better able to encourage peer support in remote groups during the current study or that, by this later stage of the pandemic, there was more familiarity, openness, and willingness to engage with peers using remote modalities, including discussion of sensitive mental health concerns in these settings.

Additionally, quantitative results of the parent RCT (forthcoming) are also important to consider when interpreting these results. Quantitative analyses revealed significant reductions in mental health outcomes (i.e., symptoms of depression, anxiety, and PTSD) for participants in in-person groups but not remote groups (results unpublished). Barriers to implementation in the remote modality, such as connectivity issues, privacy constraints, and power shortages, might help to explain the lack of effectiveness despite the psychosocial skills changes (i.e., community-building, problem-solving, and emotional regulation) reported by participants in both modalities in the current study. Numerous studies suggest that, while digital interventions present a promising approach to MHPSS service delivery, more work is needed to evaluate effectiveness and identify and address the barriers that may impede feasibility, acceptability, effectiveness, and scalability.^29^^,^^59^^,^^74^^–^^76^

### Recommendations for Integrating Community-Based Psychosocial Support Groups Into Routine Mental Health Care Services

This research reveals several key strategies for the integration of the CB-PSS groups into routine health care and social services in Colombia and similar contexts, where mental health services are challenging to provide.^27^

#### Strengthen Training and Supervision of Community Psychosocial Agents

CPAs play a pivotal role in enhancing community awareness, integrating cultural codes to foster acceptance of mental health care services, and reducing stigma.^22^ As a result, they emerge as key agents in establishing a bidirectional route, connecting with community members, and informing specialized health providers about the cultural appropriateness of integration into their practices.^4^^,^^14^^,^^77^^,^^78^

CPAs play a pivotal role in enhancing community awareness, integrating cultural codes to foster acceptance of mental health care services, and reducing stigma.

- Provide comprehensive training for CPAs in evidence-based protocols, such as PM+.
- Emphasize the importance of CPAs’ regular supervision to enhance their capabilities and prevent burnout to ensure quality and sustainable services.
- Encourage CPAs to inform specialized health providers about cultural appropriateness integration into their practices.
- Focus on capacity-building for supervisors to provide effective oversight.

#### Integrate Mental Health Care Services With Existing Primary Care Services

Collaborating within existing primary health care services, such as community service posts, telehealth, and outreach services, offers a pathway for integrating access to and adherence to specialized care when needed (e.g., psychologists, social workers, psychiatrists).^14^^,^^77^ To support such interventions, local and international nonprofit organizations can assist in coordination with local governmental institutions and providers. It is crucial to devise a plan to gradually hand over activities, promoting community ownership and long-term sustainability.^22^

- Integrate CB-PSS services into existing primary public health facilities that operate within community service posts, telehealth, or as part of mobile outreach services.
- Strengthen CPAs’ skills to effectively connect with community members, fostering awareness and reducing stigma to promote greater acceptance of mental health care services.
- Facilitate effective communication between community members and service providers for referrals to specialized care when needed (e.g., psychologists, social workers, and psychiatrists).

#### Coordinate Local Partnerships

A coordinated partnership network involving relevant actors (e.g., local governmental institutions, the private sector, nongovernmental organizations, community associations, and local leaders) operating in such contexts represents potential opportunities to advance the appropriate transition in legislation, policy, and programming. This transition should aim to establish an inclusive, equitable, and culturally appropriate mental health local system.^79^^,^^80^

- Coordinate partnerships with local governmental organizations, local institutions (nongovernmental organizations and community associations), and the private sector to guarantee progressive transference of the CB-PSS interventions to promote community ownership and sustainability.

### Lessons Learned for Practitioners

- CB-PSS groups and TSMs represent a practical approach to improve access to psychosocial support services in vulnerable communities where professionals are scarce; however, training and supervision, including peer supervision, are key to enhancing the potential and protecting the well-being of community lay providers.
- Consider opportunities to integrate traditional community practices, such as the comadreo, to facilitate community buy-in to psychosocial support services.
- Practitioners should partner with participants, community leaders, and lay providers to design and pilot strategies to overcome contextual challenges and capitalize on contextual facilitators to promote participant engagement and retention.
- Particular attention should be paid to determining if and how to ensure incentives are provided to allow participants to effectively utilize remote modalities (e.g., technological access and Internet connectivity) and in-person modalities (e.g., transportation stipends) while allowing for feasible scale-up and sustainability of CB-PSS interventions.
- More work is needed to explore the potential of implementing hybrid modalities (combining in-person and remote modalities).

### Lessons Learned for Policymakers

- Establish funding opportunities and dissemination spaces to encourage and share research on strategies and interventions for implementing CB-PSS services.
- Promote collaboration between local and scientific communities to develop evidence-based and culturally sensitive policies for people affected by violence, displacement, and life-challenging conditions.
- Support policies that promote access to the Internet and technological devices to increase the viability of implementing remote psychosocial support services interventions for all communities, including in rural and low-resource settings.
- Advocate for policies that integrate community-based services into the routine mental health care services and system to ensure the bidirectional translation of evidence-based approaches and cultural codes into care provision.

### Limitations of the Intervention and Future Research

The present study has limitations that need acknowledgment. The current study did not identify differential factors and dynamics between rural and urban areas in Quibdó that influenced the implementation and outcomes of the CB-PSS intervention. To draw comparisons between these 2 contexts, further studies should aim to replicate this and other interventions previously implemented in the region,^37^^,^^39^ specifically within rural areas. In addition, the current study reported the intensive utilization of incentives, such as transportation and Internet stipends, as well as participant meals, during the implementation of the intervention. While these supports were deemed necessary to ensure safe participation amid the COVID-19 pandemic, they also prompt inquiries regarding the feasibility of scaling up and sustaining this intervention by community actors, especially in an LMIC setting where costly incentives may not be feasible in the long term. Moreover, the training and supervision demanded substantial resources, posing challenges for sustainability. Further studies should evaluate the feasibility of implementing interventions like the CB-PSS with less intensive incentives. Optimization of supervision and training resources should be explored through utilizing national or regional online training and supervision teams. This is particularly pertinent under less favorable conditions, such as those imposed by the COVID-19 pandemic and the national strike contingencies.

As a first step, following the conclusion of this study, the results were shared with governmental agencies and partner organizations, and facilitators trained by HAI were equipped to implement the model in various settings as part of other projects, with necessary adaptations for the post-pandemic context, including a reduction in the provision of incentives. An initial phase involving 14 community groups was implemented in Baudó, Chocó, with 308 participants. During the 8 sessions, only refreshments were provided, yet attendance remained stable, indicating sustained engagement (all participants who began the intervention completed it). However, additional community-based research is needed to understand the impact of incentives often used to increase participation in interventions, especially those implemented by international organizations within communities. This research should ensure that these incentives are fair, noncoercive, and responsive to communities’ needs while also being sustainable. This aspect is important for fostering community ownership and autonomy, thereby enhancing the potential for scaling up the interventions in diverse settings.^81^^,^^82^

## CONCLUSIONS

This article examines qualitative data from a research study on a CB-PSS group intervention conducted in Quibdó, Colombia, during the COVID-19 pandemic. The CB-PSS group approach presents a unique opportunity to align with the Colombian policy framework for implementing Colombia’s Peace Accords and potential for integration into routine mental health care services. While this model is tailored to the MHPSS needs and cultural characteristics of the Quibdó population, it also demonstrates the potential for adaptation and scale-up to other communities. The data suggest promising results and opportunities for implementing CB-PSS groups in remote and in-person modalities. However, results also emphasize the need for attention to contextual barriers that can impede participant engagement and retention, especially in remote modalities. Future work is needed to develop and test innovative, culturally tailored approaches to address the barriers highlighted in this intervention and harness and capitalize on contextual opportunities.

## Notes

**Peer Reviewed**

**First published online:** January 22, 2024.

## References

[1] Deimling Johns L, Power J, MacLachlan M. Community-based mental health intervention skills: task shifting in low- and middle-income settings. Int Perspect Psychol. 2018;7(4):205–230. 10.1037/ipp0000097

[2] Votruba N, Thornicroft G. The importance of mental health in the Sustainable Development Goals. BJPsych Int. 2015;12(1):2–4. 10.1192/S2056474000000027. 29093831 PMC5619591

[3] Spanhel K, Balci S, Baumeister H, Bengel J, Sander LB. Cultural adaptation of Internet- and mobile-based interventions for mental disorders: a systematic review protocol. Syst Rev. 2020;9(1):207. 10.1186/s13643-020-01438-y. 32883367 PMC7472576

[4] Jordans MJD, Kohrt BA. Scaling up mental health care and psychosocial support in low-resource settings: a roadmap to impact. Epidemiol Psychiatr Sci. 2020;29:e189. 10.1017/S2045796020001018. 33239113 PMC7737188

[5] Registro Único de Víctimas (RUV). Unidad para las Víctimas del Conflicto. July 10, 2017. Accessed December 13, 2023. https://www.unidadvictimas.gov.co/es/registro-unico-de-victimas-ruv/37394

[6] Bonilla-Escobar FJ, Osorio-Cuellar GV, Pacichana-Quinayáz SG, Sánchez-Rentería G, Fandiño-Losada A, Gutiérrez MI. Do not forget culture when implementing mental health interventions for violence survivors. Cien Saude Colet. 2017;22(9):3053–3059. 10.1590/1413-81232017229.12982016. 28954156

[7] Castro-Camacho L, Rattner M, Quant DM, González L, Moreno JD, Ametaj A. A contextual adaptation of the unified protocol for the transdiagnostic treatment of emotional disorders in victims of the armed conflict in Colombia. Cognit Behav Pract. 2019;26(2):351–365. 10.1016/j.cbpra.2018.08.002

[8] Bonilla-Escobar FJ, Osorio-Cuéllar GV, Pacichana-Quinayaz SG, et al. Impacts of violence on the mental health of Afro-descendant survivors in Colombia. Med Confl Surviv. 2021;37(2):124–145. 10.1080/13623699.2021.1938035. 34225496

[9] Congreso de la República de Colombia. Ley No.1448: Junio 10 de 2011. Accessed December 13, 2023. https://www.unidadvictimas.gov.co/sites/default/files/documentosbiblioteca/ley-1448-de-2011.pdf

[10] Ministerio del Interior República de Colombia. Decreto de Ley No. 4634 de 2011. 9 de Diciembre de 2011. Accessed December 13, 2023. https://www.funcionpublica.gov.co/eva/gestornormativo/norma_pdf.php?i=44972

[11] Ministerio del Interior República de Colombia. Decreto Ley No. 4635 de 2011. 9 de Diciembre de 2011. Accessed December 13, 2023. https://www.funcionpublica.gov.co/eva/gestornormativo/norma_pdf.php?i=44984

[12] Congreso Nacional de la República de Colombia. Ley 1438 de 19 Enero de 2011. Accessed December 13, 2023. https://www.minsalud.gov.co/Normatividad_Nuevo/LEY%201438%20DE%202011.pdf

[13] Ministerio de Salud y Protección Social de la República de Colombia. Decreto 376 del 14 de Marzo 2022. Accessed December 13, 2023. https://www.minsalud.gov.co/Normatividad_Nuevo/Decreto%20No.%20376%20de%202022.pdf

[14] Agudelo-Hernández F, García Cano JF, Salazar Vieira LM, Vergara Palacios W, Padilla M, Moreno Mayorga B. Brechas en la atención primaria en salud mental en Chocó, Colombia: barreras y desafíos. [Gaps in primary mental health care in Chocó, Colombia: barriers and challenges]. Article in Spanish. Rev Panam Salud Publica. 2023;47:1. 10.26633/RPSP.2023.138. 37881801 PMC10597394

[15] Campo-Arias A, Ceballos-Ospino GA, Herazo E. Barriers to access to mental health services among Colombia outpatients. Int J Soc Psychiatry. 2020;66(6):600–606. 10.1177/0020764020925105. 32466709

[16] Chaskel R, Gaviria SL, Espinel Z, Taborda E, Vanegas R, Shultz JM. Mental health in Colombia. BJPsych Int. 2015;12(4):95–97. 10.1192/S2056474000000660. 29093873 PMC5618872

[17] Xiong J, Lipsitz O, Nasri F, et al. Impact of COVID-19 pandemic on mental health in the general population: a systematic review. J Affect Disord. 2020;277:55–64. 10.1016/j.jad.2020.08.001. 32799105 PMC7413844

[18] Gillies A, Hume M, Camburn M, et al. *COVID-19 in Chocó, Colombia: Learning From Grassroot Responses to the Pandemic*. Low and Middle Income Countries Research Network; 2021. Accessed December 13, 2023. 10.36399/gla.pubs.260010

[19] Sanabria-Mazo JP, Useche-Aldana B, Ochoa PP, et al. Social inequities in the impact of COVID-19 lockdown measures on the mental health of a large sample of the Colombian population (PSY-COVID study). J Clin Med. 2021;10(22):5297. 10.3390/jcm10225297. 34830579 PMC8619612

[20] World Health Organization (WHO). *Task Shifting: Rational Redistribution of Tasks Among Health Workforce Teams: Global Recommendations and Guidelines*. WHO; 2008. Accessed December 13, 2023. https://www.unaids.org/sites/default/files/media_asset/ttr_taskshifting_en_0.pdf

[21] Raviola G, Naslund JA, Smith SL, Patel V. Innovative models in mental health delivery systems: task sharing care with non-specialist providers to close the mental health treatment gap. Curr Psychiatry Rep. 2019;21(6):44. 10.1007/s11920-019-1028-x. 31041554

[22] Javadi D, Feldhaus I, Mancuso A, Ghaffar A. Applying systems thinking to task shifting for mental health using lay providers: a review of the evidence. Glob Ment Health (Camb). 2017;4:e14. 10.1017/gmh.2017.15. 29230310 PMC5719475

[23] World Health Organization (WHO). *mhGAP Intervention Guide for Mental, Neurological and Substance Use Disorders in Non-Specialized Health Settings: Mental Health Gap Action Programme (mhGAP) – Version 2.0*. WHO; 2016. Accessed December 13, 2023. https://iris.who.int/bitstream/handle/10665/250239/9789241549790-eng.pdf 27786430

[24] van’t Hof E, Sangraula M, Luitel NP, et al. Effectiveness of Group Problem Management Plus (Group-PM+) for adults affected by humanitarian crises in Nepal: study protocol for a cluster randomized controlled trial. Trials. 2020;21(1):343. 10.1186/s13063-020-04263-9. 32307009 PMC7168994

[25] World Health Organization (WHO). *Problem Management Plus (PM+): Individual Psychological Help for Adults Impaired by Distress in Communities Exposed to Adversity, WHO Generic Field-Trail Version 1.0*. WHO; 2016. Accessed December 13, 2023. https://iris.who.int/handle/10665/206417

[26] Taylor CB, Fitzsimmons-Craft EE, Graham AK. Digital technology can revolutionize mental health services delivery: the COVID‐19 crisis as a catalyst for change. Int J Eat Disord. 2020;53(7):1155–1157. 10.1002/eat.23300. 32449523 PMC7280562

[27] Kola L, Kohrt BA, Hanlon C, et al. COVID-19 mental health impact and responses in low-income and middle-income countries: reimagining global mental health. Lancet Psychiatry. 2021;8(6):535–550. 10.1016/S2215-0366(21)00025-0. 33639109 PMC9764935

[28] Cohen F, Yaeger L. Task-shifting for refugee mental health and psychosocial support: a scoping review of services in humanitarian settings through the lens of RE-AIM. Implement Res Pract. 2021;2:2633489521998790. 10.1177/2633489521998790. 37089989 PMC9978653

[29] Carter H, Araya R, Anjur K, Deng D, Naslund JA. The emergence of digital mental health in low-income and middle-income countries: a review of recent advances and implications for the treatment and prevention of mental disorders. J Psychiatr Res. 2021;133:223–246. 10.1016/j.jpsychires.2020.12.016. 33360867 PMC8801979

[30] Le PD, Eschliman EL, Grivel MM, et al. Barriers and facilitators to implementation of evidence-based task-sharing mental health interventions in low- and middle-income countries: a systematic review using implementation science frameworks. Implement Sci. 2022;17(1):4. 10.1186/s13012-021-01179-z. 35022081 PMC8756725

[31] Rattner M, James LE, Botero JF, et al. Piloting a community-based psychosocial group intervention designed to reduce distress among conflict-affected adults in Colombia: a mixed-method study of remote, hybrid, and in-person modalities during the COVID-19 pandemic. Int J Ment Health Syst. 2023;17(1):35. 10.1186/s13033-023-00597-4. 37875939 PMC10594726

[32] Ficha de caracterización de Quibdó. Departamento Nacional de Planeación. 2022. Accessed December 13, 2023. https://terridata.dnp.gov.co/index-app.html#/perfiles/27001

[33] Cazabat C, Ferrández PC. *Impacts of Displacement: Conflict and Violence in Quibdó and Caucasia, Colombia*. Internal Displacement Monitoring Centre; 2022. Accessed December 13, 2023. https://www.internal-displacement.org/sites/default/files/publications/documents/220302_IDMC_SocioeconomicImpacts_Colombia.pdf

[34] Estatuto temporal de protección-prerregistros en el sistema [Temporary statute of protection-pre-registrations in the system]. Migración Colombia. October 26, 2021. Updated December 13, 2023. Accessed December 13, 2023. https://public.tableau.com/app/profile/migraci.n.colombia/viz/EstatutoTemporaldeProteccin-Prerregistros/Pre-registrosPublic

[35] Espinel Z, Chaskel R, Berg RC, et al. Venezuelan migrants in Colombia: COVID-19 and mental health. Lancet Psychiatry. 2020;7(8):653–655. 10.1016/S2215-0366(20)30242-X. 32711697 PMC7377810

[36] Naciones Unidas. *El Paro Nacional 2021: Lecciones Aprendidas para el Ejercicio del Derecho de Reunión Pacífica en Colombia [The 2021 National Strike: Lessons Learned for the Exercise of the Right of Peaceful Assembly in Colombia.]*. Naciones Unidas; 2021. Accessed December 28, 2023. https://www.hchr.org.co/wp/wp-content/uploads/2022/05/211214-Colombia_Documento-lecciones-aprendidas-y-observaciones-Paro-Nacional-2021.pdf

[37] Bonilla-Escobar FJ, Fandiño-Losada A, Martínez-Buitrago DM, et al. A randomized controlled trial of a transdiagnostic cognitive-behavioral intervention for Afro-descendants’ survivors of systemic violence in Colombia. PLoS One. 2018;13(12):e0208483. 10.1371/journal.pone.0208483. 30532155 PMC6287825

[38] Osorio-Cuellar GV, Pacichana-Quinayáz SG, Bonilla-Escobar FJ, Fandiño-Losada A, Gutiérrez-Martinez MI. Perceptions about implementation of a Narrative Community-Based Group Therapy for Afro-Colombians victims of violence. Cien Saude Colet. 2017;22(9):3045–3052. 10.1590/1413-81232017229.00402016. 28954155

[39] Bonilla-Escobar FJ, Fandiño-Losada A, Martinez-Buitrago DM, et al. Mental health Narrative Community-Based Group Therapy in violence-displaced Afro-Colombians: a randomized controlled trial. Med Confl Surviv. 2023;39(1):28–47. 10.1080/13623699.2023.2177951. 36815261

[40] Pacichana-Quinayáz SG, Osorio-Cuéllar GV, Bonilla-Escobar FJ, Fandiño-Losada A, Gutiérrez-Martínez MI. Common elements treatment approach based on a cognitive behavioral intervention: implementation in the Colombian Pacific. Cien Saude Colet. 2016;21(6):1947–1956. 10.1590/1413-81232015216.07062015. 27276543

[41] Aranguren-Romero JP, Rubio-Castro N. Formación en herramientas terapéuticas a sobrevivientes del conflicto armado en el Pacífico colombiano: reflexividad y cuidado de sí. [A process of training the survivors of the Colombian armed conflict on the pacific coast in therapeutic skills: reflexivity and self-care]. Article in Spanish. Rev Estud Soc. 2018;66:18–29. 10.7440/res66.2018.03

[42] Kemp CG, Jarrett BA, Kwon CS, et al. Implementation science and stigma reduction interventions in low- and middle-income countries: a systematic review. BMC Med. 2019;17(1):6. 10.1186/s12916-018-1237-x. 30764820 PMC6376798

[43] Creswell JW, David Creswell J. *Research Design: Qualitative, Quantitative, and Mixed Methods Approaches*. 5th ed. Sage Publications; 2018.

[44] Sundler AJ, Lindberg E, Nilsson C, Palmér L. Qualitative thematic analysis based on descriptive phenomenology. Nurs Open. 2019;6(3):733–739. 10.1002/nop2.275. 31367394 PMC6650661

[45] Gulamani T, Uliaszek AA, Chugani CD, Rashid T. Attrition and attendance in group therapy for university students: an examination of predictors across time. J Clin Psychol. 2020;76(12):2155–2169. 10.1002/jclp.23042. 32830326 PMC7666048

[46] Pedersen GA, Sangraula M, Shrestha P, et al. Developing the group facilitation assessment of competencies tool for group-based mental health and psychosocial support interventions in humanitarian and low-resource settings. J Educ Emerg. 2021;7(2):334. 10.33682/u4t0-acde

[47] Alloh FT, Regmi P, Onche I, Teijlingen EV, Trenoweth S. Mental health in low-and middle income countries (LMICs): going beyond the need for funding. Health Perspect. 2018;17(1):12–17. 10.3126/hprospect.v17i1.20351

[48] Esponda GM, Hartman S, Qureshi O, Sadler E, Cohen A, Kakuma R. Barriers and facilitators of mental health programmes in primary care in low-income and middle-income countries. Lancet Psychiatry. 2020;7(1):78–92. 10.1016/S2215-0366(19)30125-7. 31474568

[49] Troup J, Fuhr DC, Woodward A, Sondorp E, Roberts B. Barriers and facilitators for scaling up mental health and psychosocial support interventions in low- and middle-income countries for populations affected by humanitarian crises: a systematic review. Int J Ment Health Syst. 2021;15(1):5. 10.1186/s13033-020-00431-1. 33413526 PMC7792016

[50] Santaella-Tenorio J, Bonilla-Escobar FJ, Nieto-Gil L, et al. Mental health and psychosocial problems and needs of violence survivors in the Colombian Pacific Coast: a qualitative study in Buenaventura and Quibdó. Prehosp Disaster Med. 2018;33(6):567–574. 10.1017/S1049023X18000523. 30047356

[51] Chatterjee S, Chowdhary N, Pednekar S, et al. Integrating evidence‐based treatments for common mental disorders in routine primary care: feasibility and acceptability of the MANAS intervention in Goa, India. World Psychiatry. 2008;7(1):39–46. 10.1002/j.2051-5545.2008.tb00151.x. 18458786 PMC2359726

[52] Marliana T, Keliat BA, Kurniawan K. Mental health and psychosocial support online-based services to improve elderly integrity and reduce loneliness during the pandemic Covid-19. Mal J Med Health Sci. 2022;18(Suppl 3):1–6. Accessed December 13, 2023. https://medic.upm.edu.my/upload/dokumen/202202212338021_0987.pdf

[53] Dickson K, Bangpan M. What are the barriers to, and facilitators of, implementing and receiving MHPSS programmes delivered to populations affected by humanitarian emergencies? A qualitative evidence synthesis. Glob Ment Health (Camb). 2018;5:e21. 10.1017/gmh.2018.12. 29997893 PMC6036649

[54] Schultz K, Cattaneo LB, Sabina C, Brunner L, Jackson S, Serrata JV. Key roles of community connectedness in healing from trauma. Psychol Violence. 2016;6(1):42–48. 10.1037/vio0000025

[55] Seifert A, Cotten SR, Xie B. A double burden of exclusion? Digital and social exclusion of older adults in times of COVID-19. J Gerontol B Psychol Sci Soc Sci. 2021;76(3):e99–e103. 10.1093/geronb/gbaa098. 32672332 PMC7454901

[56] República de Colombia. Ministerio de Tecnología Información y Comunicación. *Boletín Trimestral de Las TIC Tercer Trimestre de 2021 [ICT Quarterly Bulletin Third Quarter of 2021]*. Ministerio de Tecnología Información y Comunicación; 2022. Accessed December 13, 2023. https://colombiatic.mintic.gov.co/679/w3-article-198842.html

[57] Almathami HKY, Win KT, Vlahu-Gjorgievska E. Barriers and facilitators that influence telemedicine-based, real-time, online consultation at patients’ homes: systematic literature review. J Med Internet Res. 2020;22(2):e16407. 10.2196/16407. 32130131 PMC7059083

[58] Ibragimov K, Palma M, Keane G, et al. Shifting to tele-mental health in humanitarian and crisis settings: an evaluation of Médecins Sans Frontières experience during the COVID-19 pandemic. Confl Health. 2022;16(1):6. 10.1186/s13031-022-00437-1. 35164807 PMC8845383

[59] Naslund JA, Shidhaye R, Patel V. Digital technology for building capacity of nonspecialist health workers for task sharing and scaling up mental health care globally. Harv Rev Psychiatry. 2019;27(3):181–192. 10.1097/HRP.0000000000000217. 30958400 PMC6517068

[60] Elshazly M, Alam ANMM, Ventevogel P. Field-level coordination of mental health and psychosocial support (MHPSS) services for Rohingya refugees in Cox’s Bazar. Intervention (Amstelveen). 2019;17(2):212–216. 10.4103/INTV.INTV_38_19

[61] Kamali M, Munyuzangabo M, Siddiqui FJ, et al. Delivering mental health and psychosocial support interventions to women and children in conflict settings: a systematic review. BMJ Glob Health. 2020;5(3):e002014. 10.1136/bmjgh-2019-002014. 32201624 PMC7073823

[62] Serrata JV, Macías RL, Rosales A, Rodríguez R, Perilla JL. A study of immigrant Latina survivors of domestic violence: becoming líderes comunitarias (community leaders). In: Espin OM, Dottolo AL, eds. *Gendered Journeys: Women, Migration and Feminist Psychology*. Palgrave Macmillan; 2015:206–223.

[63] Serrata JV, Rodriguez R, Castro JE, Hernandez-Martinez M. Well-being of Latina survivors of intimate partner violence and sexual assault receiving trauma-informed and culturally-specific services. J Fam Violence. 2020;35(2):169–180. 10.1007/s10896-019-00049-z

[64] Serrata JV, Macias RL, Rosales A, Hernandez-Martinez M, Rodriguez R, Perilla JL. Expanding evidence-based practice models for domestic violence initiatives: a community-centered approach. Psychol Violence. 2017;7(1):158–165. 10.1037/vio0000051

[65] Sultana A, Sharma R, Hossain MM, Bhattacharya S, Purohit N. Burnout among healthcare providers during COVID-19: challenges and evidence-based interventions. Indian J Med Ethics. 2020;05(04):308–311. 10.20529/IJME.2020.73. 34018959

[66] Soisangwarn A, Wongwanich S. Promoting the reflective teacher through peer coaching to improve teaching skills. Procedia Soc Behav Sci. 2014;116:2504–2511. 10.1016/j.sbspro.2014.01.601

[67] Murray LK, Dorsey S, Bolton P, et al. Building capacity in mental health interventions in low resource countries: an apprenticeship model for training local providers. Int J Ment Health Syst. 2011;5(1):30. 10.1186/1752-4458-5-30. 22099582 PMC3284435

[68] Abujaber N, Vallières F, McBride KA, et al. Examining the evidence for best practice guidelines in supportive supervision of lay health care providers in humanitarian emergencies: a systematic scoping review. J Glob Health. 2022;12:04017. 10.7189/jogh.12.04017. 35265328 PMC8876157

[69] Luzano JGC, Mordeno IG. Assessing the efficacy of the problem management plus (PM+) in improving the mental health of conflict-exposed individuals. Curr Psychol. 2023:1–17. 10.1007/s12144-023-05326-1

[70] Bryant R, Bawaneh A, Awwad M, et al. *End Report on Process and Outcomes of PM+ Implementation in Jordan*. Scaling Up Psychological Interventions with Syrian Refugees; 2022. Accessed December 13, 2023. https://strengths-project.eu/wp-content/uploads/2023/03/D4.2_-D4.3-End-report-Jordan.pdf

[71] Pedersen GA, Lakshmin P, Schafer A, et al. Common factors in psychological treatments delivered by non-specialists in low- and middle-income countries: manual review of competencies. J Behav Cogn Ther. 2020;30(3):165–186. 10.1016/j.jbct.2020.06.001. 34308387 PMC8297897

[72] Al-Tamimi SAGA, Leavey G. Community-based interventions for the treatment and management of conflict-related trauma in low-middle income, conflict-affected countries: a realist review. J Child Adolesc Trauma. 2022;15(2):441–450. 10.1007/s40653-021-00373-x. 35600528 PMC9120315

[73] Castro-Camacho L, Barlow DH, García N, et al. Effects of a contextual adaptation of the unified protocol in multiple emotional disorders in individuals exposed to armed conflict in Colombia. JAMA Psychiatry. 2023;80(10):991–999. 10.1001/jamapsychiatry.2023.2392. 37466983 PMC10357366

[74] Naslund JA, Marsch LA, McHugo GJ, Bartels SJ. Emerging mHealth and eHealth interventions for serious mental illness: a review of the literature. J Ment Health. 2015;24(5):321–332. 10.3109/09638237.2015.1019054. 26017625 PMC4924808

[75] Naslund JA, Aschbrenner KA, Araya R, et al. Digital technology for treating and preventing mental disorders in low-income and middle-income countries: a narrative review of the literature. Lancet Psychiatry. 2017;4(6):486–500. 10.1016/S2215-0366(17)30096-2. 28433615 PMC5523650

[76] Arjadi R, Nauta MH, Chowdhary N, Bockting CLH. A systematic review of online interventions for mental health in low and middle income countries: a neglected field. Glob Ment Health (Camb). 2015;2:e12. 10.1017/gmh.2015.10. 28596860 PMC5269629

[77] Bolton P, West J, Whitney C, et al. Expanding mental health services in low- and middle-income countries: a task-shifting framework for delivery of comprehensive, collaborative, and community-based care. Glob Ment Health (Camb). 2023;10:e16. 10.1017/gmh.2023.5. 37854402 PMC10579648

[78] Jordans MJD, Luitel NP, Kohrt BA, et al. Community-, facility-, and individual-level outcomes of a district mental healthcare plan in a low-resource setting in Nepal: a population-based evaluation. PLoS Med. 2019;16(2):e1002748. 10.1371/journal.pmed.1002748. 30763321 PMC6375569

[79] Inter-Agency Standing Committee (IASC). *The Mental Health and Psychosocial Support Minimum Service Package*. IASC; 2022. Accessed December 13, 2023. https://interagencystandingcommittee.org/sites/default/files/migrated/2023-01/IASC%20MHPSS%20Minimum%20Service%20Package.pdf 10.1080/09540261.2022.2147420 36502397

[80] World Health Organization (WHO). *World Mental Health Report: Transforming Mental Health for All*. WHO; 2022. Accessed December 13, 2023. https://iris.who.int/bitstream/handle/10665/356119/9789240049338-eng.pdf

[81] Reid N, Buchman D, Brown R, Pedersen C, Kozloff N, Stergiopoulos V. The acceptability of financial incentives to support service engagement of adults experiencing homelessness and mental illness: a qualitative study of key stakeholder perspectives authorship. Adm Policy Ment Health. 2022;49(6):1060–1071. 10.1007/s10488-022-01217-y. 36071341

[82] Giles EL, Robalino S, Sniehotta FF, Adams J, McColl E. Acceptability of financial incentives for encouraging uptake of healthy behaviours: a critical review using systematic methods. Prev Med. 2015;73:145–158. 10.1016/j.ypmed.2014.12.029. 25600881

